# Preparation and Antioxidant Properties of Germinated Soybean Protein Hydrolysates

**DOI:** 10.3389/fnut.2022.866239

**Published:** 2022-05-12

**Authors:** Qianhui Qi, Guohua Zhang, Wei Wang, Faizan Ahmed Sadiq, Yu Zhang, Xue Li, Qihe Chen, Qile Xia, Xinquan Wang, Yougui Li

**Affiliations:** ^1^College of Life Sciences, Shanxi University, Taiyuan, China; ^2^State Key Laboratory for Managing Biotic and Chemical Threats to the Quality and Safety of Agro-products, Institute of Agro-product Safety and Nutrition, Zhejiang Academy of Agricultural Sciences, Hangzhou, China; ^3^School of Food Science and Technology, Jiangnan University, Wuxi, China; ^4^Key Laboratory of Information Traceability for Agricultural Products, Ministry of Agriculture and Rural Affairs of China, Hangzhou, China; ^5^College of Biosystems Engineering and Food Science, Zhejiang University, Hangzhou, China

**Keywords:** antioxidative activity, germinated soybean, isolation, purification, protein hydrolysates

## Abstract

In this study, soybeans during different germination stages were described and compared with regard to morphology, water content, protein, amino acids, and isoflavones. The optimal conditions for the hydrolysis of proteins obtained from germinated soybeans were determined using the response surface methodology. Gel filtration chromatography was used to separate germinated soybean protein hydrolysates after ultrafiltration, whereas 2,2-Diphenyl-1-picrylhydrazyl (DPPH), ABTS^•+^, and FRAP assays were used to assess the antioxidant activity of different fractions. Findings of this study revealed that protein and isoflavone contents were high in soybean at 24 h following germination (the bud was about 0.5–1 cm). The proteins from germinated soybeans were hydrolyzed and separated into five fractions (G1–G5) and evaluated in terms of their molecular weight and antioxidant activity. Interestingly, the antioxidant activity was found to be higher in germinated soybean protein hydrolysates than in other soybean protein hydrolysates derived from soybean meal protein. This suggests that germination can effectively improve the utilization rate of soybean proteins. The antioxidant activity of G3 was best among G1–G5. The results obtained in this study demonstrate that germination for 24 h when the bud length is about 0.5–1 cm can be applied as a special pretreatment of plant seeds in the development of germinated foods. These findings can be used to identify the structure of the potential antioxidative hydrolysates for their possible exploitation in functional foods.

## Introduction

Soybean (*Glycine max L*.), one of the most important food crops in the world, is known for its high-quality plant-derived oils, high crude protein content (40%), fats (20%), and carbohydrates (35%) (1–3). Over the past two decades, bioactive food protein hydrolysates and peptides have gained great research interest due to their physiological functions, such as antioxidant (4, 5), antibacterial (5), anticancer (6), antidiabetic (7), and antihypertensive properties (4, 8, 9). Besides, they play an important role in immune regulation (10). β-Conglycinin (βCG, 7S) and glycinin (11S) are the two major proteins of soybean which account for up to 90% of the total protein content. Soybean is a rich source of biologically active peptides with a wide array of physiological functions (11). Soybean peptides can be generated from soybean meal protein following oil extraction by enzymatic hydrolysis (12, 13). However, this method has many disadvantages, such as low hydrolysis yield and the production of high-molecular-weight peptides (9). Germination is a common food processing method applied for treating plant seeds (14). In this process, the respiratory metabolism of plant seeds is enhanced and endogenous enzymes are activated, which accelerate and bring changes in the physiological and biochemical metabolism of the seeds (15). Germination reportedly enhances the bioactive properties of soybeans and induces a substantial increase in many biologically important compounds, such as oestrogenic compounds, saponin, and almost all phytosterols (16, 17).

Studies have shown that germinated plant seeds may have a higher nutritional value. Germinated soybeans also show a dramatic change in the content of free and total amino acids during germination, where there is a significant increase in free amino acids and a slight decrease in the oleic acid, linoleic acid, and linolenic acid contents (18). The anti-inflammatory effect of the soybean extracts obtained from germinated soybean sprouts was found to be higher than the extracts obtained at the seed stage (19, 20). Thus, germinated soybeans are of high nutritional quality with more biologically active components and better palatability. In a recent study, González-Montoya *et al*. (21) investigated the potential of peptides obtained from germinated soybeans to modulate postprandial glycemic response through inhibition of dipeptidyl peptidase IV (DPP-IV), intestinal α-glucosidases, and salivary α-amylase. It has been reported that the potential of germinated soybean peptides (5–10 and <5 kDa fragments) derived from soybean germinated for 6 days to inhibit α-glucosidases and α-amylase, as well as antiproliferative and anti-inflammatory effects were generally higher in 5–10 kDa fractions (20). The physiological activity of hydrolysates is related to their molecular weight, peptide chain length, amino acid composition, structure, and purity (22, 23). In general, low-molecular-weight (1–5 kDa) peptides and those with high purity show higher antioxidant activity than protein hydrolysate fractions of other molecular weights (24). However, past studies about germinated soybean protein hydrolysates (GSPH) were prepared from soybean germinated for 6 days (20, 21, 25, 26), and there was no study on the preparation of bioactive protein hydrolysates during other timing of germination. The aim of this study was to assess the antioxidant potential of protein hydrolysates obtained from germinated (germination period of 24 h) soybeans.

Germinated soybeans were used as raw material for the GSPH, where the nutritional status of soybeans during different stages of germination was monitored to find out the optimal stage of germination using response surface methodology (RSM). *In vitro* trials were performed to purify GSPH and to determine their antioxidant potential. This study lays a foundation for future research on GSPH and helps to understand the significance of GSPH as bio-functional food ingredients.

## Materials and Methods

### Chemicals and Reagents

Soybeans were purchased from the China Resources Vanguard market (Hangzhou, China) in September 2020. A standard 16 amino acid mixture was purchased from Hitachi Company (Tokyo, Japan). Standards for the quantitative determination of six isoflavones [i.e., daidzin, glycitin, genistein, glycitein, daidzein, and genistin (≥98.0%)] and molecular weight determination (L-reduced glutathione (GSH), cytochrome C, aprotinin, bacitracin, Gly–Gly–Tyr–Arg, and Gly–Gly–Gly) were purchased from Shanghai Yuanye Bio-Chem Technology Co., Ltd. Acetonitrile and DPPH (>97.0%) were purchased from Sigma Company (St. Louis, USA) and were of high-performance liquid chromatography (HPLC) grade. Other chemicals used in this study were of analytical grade. Total Antioxidant Capacity Kit (FRAP) was purchased from Nanjing Jiancheng Biological Engineering Research Institute Co. Ltd. A commercial enzyme preparation containing papain (80%) and amylase (20%) was purchased from Nanning Donghenghuadao Biotechnology Co., Ltd. Normal soybean protein hydrolysates (NSPH) were purchased from Inner Mongolia Keran Biological High-tech Co., Ltd.

### Preparation of Germinated Soybean

**Figure 1:**
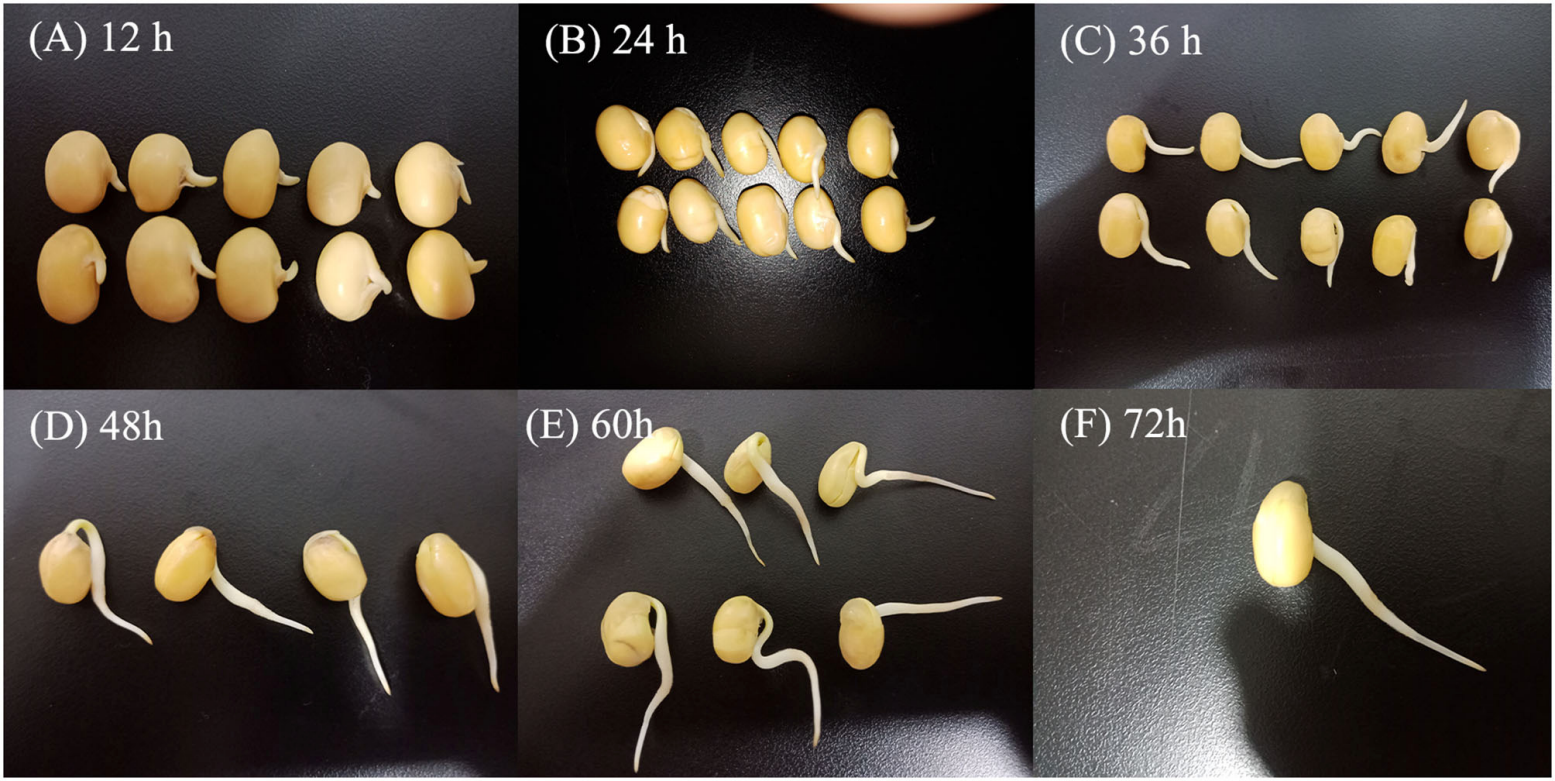
Morphological changes of soybean in different germination stages. Germination for 12 h **(A)**, germination for 24 h **(B)**, germination for 36 h **(C)**, germination for 48 h **(D)**, germination for 60 h **(E)**, and germination for 72 h **(F)**.

Yellow soybean seeds were prepared by soaking in distilled water for 12 h at room temperature. The germination process was performed in an automatized germination chamber (CB-A360B, Foshan, China) in the dark at 30°C with 10 s of irrigation every 1 h (27). The germinated seeds were harvested after 12, 24, 36, 48, 60, and 72 h (Figure 1).

The length of soybean buds was determined by the following rule: 100 soybeans were randomly selected at a certain time, and if more than 80% of the bud length was between 0.0 and 0.5, 0.5–1.0, 1.0–2.0, 2.0–3.0, and 3.0–4.0 cm, the bud length of soybean at this germination time was considered in the corresponding length ranges. Following germination, samples were homogenized and kept frozen at −20 °C until further use. Soybeans germinated at 0 h (i.e., the soybeans were soaked for 12 h) were used as control.

### Determination of Moisture, Protein, and Amino Acid Content

A total of 5.000 g of soybeans were placed in an air-blast drying oven at 105°C for 4 h. After cooling, the weight of soybeans was recorded. Then the soybeans were placed back into the air-blast drying oven and weighed every 30 min till they reached a constant weight. The moisture content was calculated based on the difference in quality before and after drying.

The protein content was determined using the Kjeldahl method (28). The soybeans (0.50 g) were digested in a digestion flask with sulfuric acid (10 ml), followed by the addition of 7.0 g of potassium sulfate and copper sulfate. The soybeans were heated at 420°C using a heating block. Then, the soybean digestive solution was boiled for a further 60 min. Next, the soybean digestive solution was cooled by cautiously adding 90 ml of water. Of note, 20 ml of the digestive solution was taken in Kjeltec Analyzer (FOSS 2300, Beijing, China) for titration. The following formula was used to calculate the protein contents:


$$\begin{array}{l} X=\frac{\left(V_{1}-V_{2}\right)\times c\times 0.0140}{m\times V_{3}/V_{4}}\times 100\times F\; \end{array}$$

where X is the protein content of the sample (%), V_1_ is the volume of hydrochloric acid standard solution that expend by sample solution (ml), V_2_ is the volume of hydrochloric acid standard solution that expend by reagent blank solution (ml), *c* is the concentration of the hydrochloric acid standard solution (mol/L), 0.0140 is the weight of nitrogen equivalent to 1.0 ml hydrochloric acid standard solution (1.000 mol/L) (g), m is the weight of the sample (g), V_3_ is the volume of the digestive solution that is taken (ml), V_4_ is the total volume of the digestive solution (ml), and F is the conversion factor (6.25).

The amino acid content was determined using an amino acid analyzer (Hitachi LA8080, Tokyo, Japan) after hydrochloric acid hydrolysis. The acid hydrolysis was carried out by assessing 0.200 g of soybeans in an ampoule with 6 mol/L hydrochloric acid solution, followed by vacuuming and sealing the ampoule on the alcohol lamp. The soybeans were heated at 110°C using the air-blast drying oven for 22 h. After cooling, it was transferred to a volumetric flask and diluted into deionized water until the volume reached 100 ml. After filtration, 5 ml of the solution was taken to a round bottom flask and evaporated using a rotary evaporator under vacuum at 40°C, followed by the addition of 5 ml of pH 2.2 sodium citrate buffer. Finally, the solution was filtered through a 0.45 μm filtration membrane before being subjected to the amino acid analyzer.

### Determination of Isoflavones

The method described by XU (29) was used with appropriate modifications to detect isoflavones in soybeans. Homogenates of the samples (1.00 g each) were put into conical flasks with 30 ml of 70% methanol. After ultrasonic treatment for 30 min, the samples were shaken for 2 h in the thermostatic oscillator (60°C). When the conical flasks were cooled to room temperature, the volume was adjusted with 70% methanol to 50.0 ml and centrifuged at 2,795 × *g* for 10 min. The supernatant was filtered through a 0.45-μm filtration membrane before being subjected to HPLC.

Isoflavones were separated using the Capcell PAK MG C18 column (250 × 4.6 mm i.d., 5 μm, Shiseido, Japan) with a mobile phase of 0.1% aqueous phosphoric acid (A) and acetonitrile (B). The gradient elution conditions were as follows: 18% phase B from 0 to 5 min, 18–32% phase B from 5 to 6 min, and then a holding time of 6–13 min; 32%−30% B from 13 to 14 min, and then a holding time of 2 min; 30–80% phase B from 16 to 17 min, and then a holding time of 3 min; 80–18% phase B from 20 to 21 min followed by a holding time of 21–25 min. The flow rate was set at 1.0 ml/min. The injection volume was 10 μl. The temperature of the column was set at 34°C. The UV detection wavelength was 260 nm.

### Preparation and Optimization of the GSPH

A single-factor test of enzymatic hydrolysis was employed in this study using a commercial enzyme preparation containing papain (80%) and amylase (20%). The effects of three factors, including enzymolysis time (X_1_), dosages of proteinase (X_2_), and the ratio of material to liquid (X_3_), were investigated. The RSM coupled with the Box-Behnken design was utilized to obtain optimal process parameters for enzymatic hydrolysis. With the hydrolysis degree (DH) as the response value, each factor corresponded to lower (−1) and higher (+1) values (Table 1). Three-factor, three-level experiments were performed, and the DH was taken as the response value. The determination of the DH was based on the method described by Lin (30).

**Table 1:** Box-Behnken design matrix with matrix experimental results of the DH and analysis of variance for the response surface model.

**Box-Behnken design**
**Standard order**	**X_**1**_ Enzymolysis time (h)**	**X_**2**_ Dosages of proteinase (%)**	**X_**3**_ Ratio for material and liquid**	**Y Degree of hydrolysis (%)**
1	3(-1)	1(-1)	1:10(0)	31.66
2	5(+1)	1(-1)	1:10(0)	39.41
3	3(-1)	3(+1)	1:10(0)	50.76
4	5(+1)	3(+1)	1:10(0)	57.41
5	3(-1)	2(0)	1:5(-1)	28.48
6	5(+1)	2(0)	1:5(-1)	41.37
7	3(-1)	2(0)	1:15(+1)	45.37
8	5(+1)	2(0)	1:15(+1)	48.50
9	4(0)	1(-1)	1:5(-1)	20.52
10	4(0)	3(+1)	1:5(-1)	42.03
11	4(0)	1(-1)	1:15(+1)	34.56
12	4(0)	3(+1)	1:15(+1)	51.41
13	4(0)	2(0)	1:10(0)	40.15
14	4(0)	2(0)	1:10(0)	41.66
15	4(0)	2(0)	1:10(0)	41.65
16	4(0)	2(0)	1:10(0)	38.24
17	4(0)	2(0)	1:10(0)	41.44
**ANOVA**						
**Source**	**Sum of squares**	**Degree of freedom**	**Mean square**	***F*** **value**	***P*****-value Prob** **>** **F**	**Remark**
Model	1256.53	9	139.61	105.43	<0.0001	***
A	115.69	1	115.69	87.36	<0.0001	***
B	711.70	1	711.70	537.43	<0.0001	***
C	281.27	1	281.27	212.40	<0.0001	***
AB	0.30	1	0.30	0.23	0.6463	
AC	23.82	1	23.81	17.99	0.0038	**
BC	5.41	1	5.41	4.09	0.0829	
A^2^	67.05	1	67.05	50.63	0.0002	***
B^2^	0.15	1	0.15	0.12	0.7425	
C^2^	57.30	1	57.30	43.27	0.0003	***
Residual	9.27	7	1.32			
Lack of Fit	0.57	3	0.19	0.09	0.9628	
Pure Error	8.69	4	2.17			
Cor Total	1265.8	16				
**Credibility analysis of the regression equations**							
**Index**	**Standard deviation**	**Mean**	**Coefficient of variation (%)**	***R* ^2^**	**Adjusted** **R^2^**	**Predicted** **R^2^**	**Adequacy precision**
R	1.15	40.86	2.82%	99.27%	98.33%	98.20%	22.78

***Significant at P < 0.001; **Significant at P < 0.01.

The soybeans were germinated for 24 h before they were homogenized. The homogenate was mixed with distilled water according to the material to liquid ratio of 1:10. After mixing well, the mixture (30 g) was put into a conical flask with the compound protease (3% of the dry matter weight) and was stirred for 5 h at 50°C in a magnetic stirrer. Then the conical flask was put into boiling water for 10 min to end the enzymatic hydrolysis, followed by centrifugation at 2,795 × *g* for 5 min. The supernatant was freeze-dried and then stored at −20°C before use.

### Separation and Purification of GSPH

The GSPH obtained in 2.4 was redissolved and then filtered using a membrane (Thermo Fisher Scientific, Waltham, USA) with a molecular weight cutoff of 3 kDa. The ultrafiltrate (<3 kDa) was collected, freeze-dried, and then stored at −20°C until further analysis.

**Figure 2:**
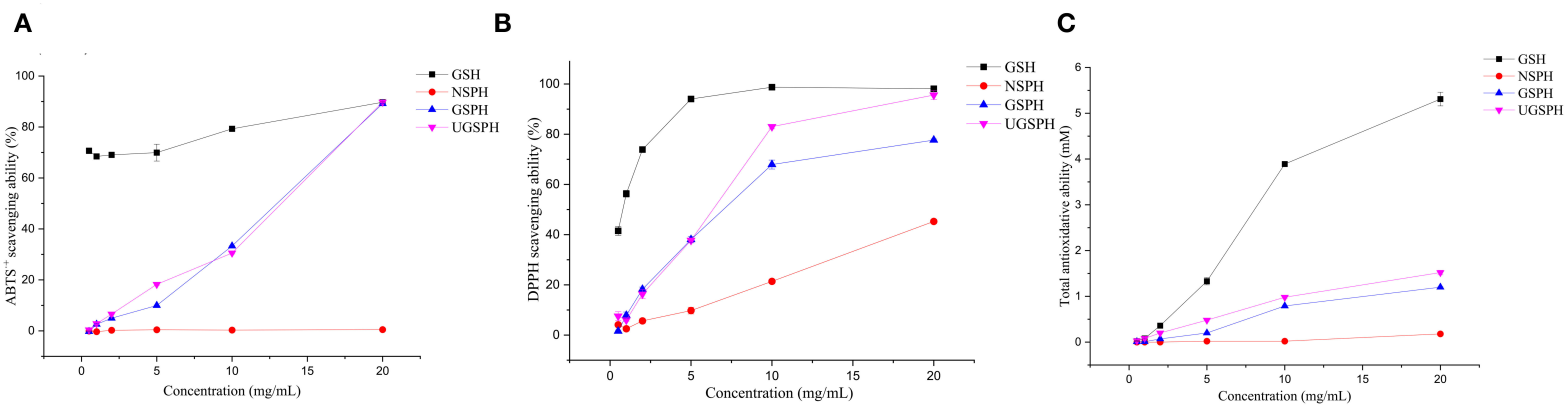
Antioxidant capacities of normal soybean protein hydrolysates (NSPH), germinated soybean protein hydrolysates (GSPH), and ultrafiltration germinated soybean protein hydrolysates (UGSPH). ABTS^•+^ scavenging ability **(A)**, DPPH scavenging ability **(B)**, and total antioxidative ability **(C)**.

The Sephadex G-15 gel was used to separate the hydrolysates collected in the previous step (GSPH after ultrafiltration, UGSPH). A certain amount of Sephadex G-15 gel dry powder was weighed and made fully swollen. Then it was loaded into the column (1.6 × 100 cm). In case of the appearance of bubbles in the gel chromatography column, the gel was reloaded, and the gel was balanced with eluent 3 times before loading samples. The separation conditions were as follows: the loading concentration was 100 mg/ml and the volume of the loading sample was 1 ml. The mobile phase was deionized water, and the flow rate was 0.5 ml/min. The detection wavelength was 280 nm. Each peak in the chromatogram (Figure 2A) was considered one fraction of UGSPH. Every fraction was collected and freeze-dried to determine the molecular weight distribution and *In vitro* antioxidant activity.

### Determination of the Molecular Weight

The sample (10 mg) was accurately weighed and diluted into deionized water until the volume reached 10 ml. The molecular weight distribution of protein hydrolysates was carried out using gel filtration chromatography with HPLC. Separation work was performed using a TSK gel G2000SWXL column (7.8 mm I. D. × 30 cm, 5 μm, Japan), with a mobile phase of 0.1% aqueous trifluoroacetic acid (A) and acetonitrile (B). The gradient elution was A: B = 55:45 (v/v). The flow rate was set at 0.5 ml/min. The injection volume was 10 μl. The temperature of the column was set at 30°C. The UV detection wavelength was 220 nm. The calibration curves were drawn using cytochrome C (Mw = 12, 400 Da), aprotinin (Mw = 6, 512 Da), bacitracin (Mw = 1, 423 Da), Gly–Gly–Tyr–Arg (Mw = 451 Da), and Gly–Gly–Gly (Mw = 189 Da). The *X*-axis of the calibration curve was the retention time, and the *Y*-axis was the lg value of relative molecular mass (lg Mw) of the above standards.

### Determination of Antioxidative Activity

#### ABTS^•+^ Free Radical Scavenging Ability

The ABTS^•+^ radical scavenging assay was performed following the protocol previously described (31). ABTS^•+^ (100.0 mg) and potassium persulfate (17.2 mg) were accurately measured, and the volume was adjusted to 50.0 ml with distilled water. After shaking, the ABTS^•+^ stock solution was placed in the dark at room temperature for 24 h. A certain volume of ABTS^•+^ stock solution was taken and diluted 40–50 times with 95% ethanol. After 30 min at room temperature, the absorbance was (0.70 ± 0.02) at 734 nm. At this stage, the mixture was used as ABTS^•+^ working solution. Samples of different concentrations, 0.5–20.0 mg/ml, were mixed with ABTS^•+^ working solution, respectively, and the mixtures were kept in the dark. The blanks and control samples were set. These mixtures were allowed to stand at 37°C for 5 min, and the absorbance was measured at 734 nm. GSH (0.5–20.0 mg/ml) was used as the positive control. The formula for calculating the free radical scavenging activity was as follows:


$$\begin{array}{l} P\left(scavenging\;rate\;\%\right)=\left(\frac{A_{blank}-A_{sample}}{A_{blank}}\right)\times 100\%\; \end{array}$$

#### DPPH Free Radical Scavenging Ability

DPPH (2.5 mg) was weighed and added into anhydrous ethanol and allowed to fully dissolve in the dark and ultrasonic environment. Next, anhydrous ethanol was used to make a volume of 50.0 ml with a concentration of 50.0 μg/ml. Samples (100 μl) of different concentrations (0.5–20.0 mg/ml and 100 μl DPPH working solution) were mixed and protected from light, and blanks and control sample were set. These mixtures were allowed to stand at room temperature for 30 min, and the absorbance was measured at 517 nm (32). GSH (0.5–20.0 mg/ml) was used as the positive control. The result can be calculated as follows:


$$\begin{array}{l} P\left(scavenging\;rate\;\%\right)=\left(1-\frac{A_{sample}-A_{control}}{A_{blank}}\right)\times 100\%\; \end{array}$$

#### Total Antioxidant Activity

The Total Antioxidant Capacity Kit (FRAP) (Nanjing Jiancheng Biological Engineering Research Institute, China) was used to determine the total antioxidant activity. A calibration standard curve of Trolox was used to calculate the total antioxidant activity with a unit of the concentration of FeSO_4_ standard solution (mM).

### Statistical Analysis

Each experimental procedure was performed three times, and data were expressed as the mean ± SD. Statistical analyses were conducted by one-way analysis of variance using IBM SPSS 20 (SPSS Inc., Chicago, USA). Duncan's test was employed to analyze the significance for multiple groups. The *P*-value < 0.05 was considered statistically significant.

## Results and Discussions

### Changes in Nutrient Composition

**Figure 3:**
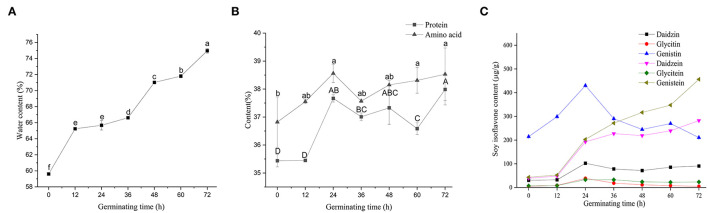
Changes of nutrients in soybean with different germination times. Water content **(A)**, protein and amino acid contents **(B)**, and soy isoflavones contents **(C)**. All results are expressed as the mean ± SD. Differences were analyzed using the Duncan's test. Groups without the same superscript letters were statistically different (*P* < 0.05).

Figure 3 shows variations in the nutritional status of soybeans during germination. Soybeans, after 12 h soaking in water, attained favorable internal environmental conditions for later germination. The protein content of non-germinated soybean was 35.43%, which was reduced during germination and early growth of the seedlings as a result of the high activity of proteases (33). The content of soybean protein showed an upward trend during germination; however, the soybean protein content remained statistically stable until 24 h following germination (Figure 3B). At 24 h following germination, there was an increase (6.32%) in soybean protein content compared to 0 h, and then a decrease (2.89%) was observed at 60 h compared to 24 h. It has been reported that soybean germination for 18 h activates endogenous proteases involved in the cleavage of storage soybean proteins and the production of bioactive peptides with antioxidant and anti-inflammatory activities (34, 35). In the beginning of seed germination, endogenous proteases stored in protein may be activated. During the enzymatic hydrolysis, these endogenous proteases can cooperate with exogenous protease to improve the efficiency of enzymatic hydrolysis. As a result, germination had positive enhancement effects on bioactive compounds in beans (36).

Amino acid composition of soybean at different germination states (all in dry matter) and their significant markers are presented in Table 2. Cys and Trp cannot be determined by acid hydrolysis and therefore were not included in the analysis. Asn and Gln were converted to Asp and Glu after the hydrolysis, respectively. Therefore, the Asx and Glx presented in Table 2 include Asp+Asn and Gln+Glu, respectively. The total amino acid content in germinated soybean increased with an increase in germination time. A total of 34 proteins related to amino acid metabolism have been reported to be involved in the process of soybean germination (27). The highest essential amino acids (EAA)/total amino acids (TAA) ratio was 0.36 at 36 h of germination, and EAA/non-essential amino acid (NEAA) ratio was 0.56. The glutamic acid content was highest followed by proline and aspartic acid. Germination in darkness has been reported as a useful method to increase the number of amino acids and thus the nutritional status of soybean products, which is congruent with our findings (37). These results show that soybean germination is associated with an increase in threonine (40.13%), aspartic acid (36.36%), and glutamic acid (33.45%), and a decrease in proline (64.66%) and histidine (18.32%), as well as the least change in the content of methionine, compared to non-germinated soybeans. Mostafa et al. (38) reported an increasing trend in different amino acid components with germination compared with the contents of the same amino acids in dry seeds. A decrease in proline content is in line with the observation of Yang et al. (39).

**Table 2:** The amino acid content in soybean with different germination time (in dry matter, g/100 g).

**Amino acid**	**Germination time**						
	**0 h**	**12 h**	**24 h**	**36 h**	**48 h**	**60 h**	**72 h**
Asx	3.52 ± 0.07^b^	4.41 ± 0.01^ab^	4.80 ± 0.21^a^	3.96 ± 0.81^ab^	4.69 ± 0.05^a^	4.46 ± 0.58^ab^	3.78 ± 0.60^ab^
Thr^&^	1.52 ± 0.04^b^	1.52 ± 0.02^b^	1.57 ± 0.17^b^	1.83 ± 0.39^ab^	1.49 ± 0.10^b^	1.68 ± 0.27^ab^	2.13 ± 0.29^a^
Ser	1.91 ± 0.04^b^	2.09 ± 0.04^ab^	2.25 ± 0.08^ab^	2.14 ± 0.07^a^	2.21 ± 0.09^a^	2.28 ± 0.10^a^	2.24 ± 0.24^a^
Glx	5.74 ± 0.14^c^	7.04 ± 0.10^b^	7.41 ± 0.06^ab^	7.33 ± 0.28^ab^	7.26 ± 0.07^b^	7.28 ± 0.06^b^	7.66 ± 0.18^a^
Gly	1.56 ± 0.03^b^	1.68 ± 0.02^a^	1.70 ± 0.00^a^	1.70 ± 0.00^a^	1.70 ± 0.01^a^	1.68 ± 0.01^a^	1.71 ± 0.03^a^
Ala	1.59 ± 0.04^c^	1.78 ± 0.02^b^	1.84 ± 0.00^ab^	1.84 ± 0.00^a^	1.86 ± 0.01^a^	1.87 ± 0.02^a^	1.86 ± 0.04^a^
Val^&^	1.62 ± 0.03^c^	1.80 ± 0.02^b^	1.84 ± 0.02^ab^	1.85 ± 0.00^a^	1.85 ± 0.01^ab^	1.85 ± 0.01^ab^	1.86 ± 0.03^a^
Met^&^	0.28 ± 0.02^a^	0.23 ± 0.01^b^	0.27 ± 0.01^a^	0.28 ± 0.01^a^	0.28 ± 0.01^a^	0.27 ± 0.00^a^	0.28 ± 0.02^a^
Ile^&^	1.60 ± 0.04^b^	1.76 ± 0.02^a^	1.76 ± 0.02^a^	1.77 ± 0.01^a^	1.76 ± 0.00^a^	1.77 ± 0.01^a^	1.78 ± 0.03^a^
Leu^&^	2.65 ± 0.06^b^	3.10 ± 0.03^a^	3.12 ± 0.01^a^	3.10 ± 0.02^a^	3.11 ± 0.02^a^	3.11 ± 0.02^a^	3.14 ± 0.06^a^
Tyr	1.13 ± 0.02^ab^	1.11 ± 0.00^ab^	1.12 ± 0.05^ab^	1.06 ± 0.04^b^	1.09 ± 0.01^ab^	1.12 ± 0.05^ab^	1.17 ± 0.01^a^
Phe^&^	1.90 ± 0.04^b^	2.10 ± 0.02^a^	2.09 ± 0.00^a^	2.08 ± 0.02^a^	2.08 ± 0.02^a^	2.09 ± 0.01^a^	2.10 ± 0.04^a^
Lys^&^	2.26 ± 0.06^c^	2.73 ± 0.02^a^	2.65 ± 0.02^ab^	2.60 ± 0.01^b^	2.65 ± 0.02^ab^	2.66 ± 0.03^ab^	2.63 ± 0.04^b^
His	1.31 ± 0.06^a^	1.08 ± 0.00^b^	1.07 ± 0.00^b^	1.07 ± 0.00^b^	1.07 ± 0.01^b^	1.08 ± 0.01^b^	1.09 ± 0.03^b^
Arg	2.75 ± 0.07^d^	3.06 ± 0.01^abc^	3.04 ± 0.04^abc^	2.96 ± 0.02^c^	2.98 ± 0.00^bc^	3.09 ± 0.07^ab^	3.16 ± 0.07^a^
Pro	5.49 ± 0.16^a^	2.07 ± 0.04^b^	2.04 ± 0.03^b^	2.01 ± 0.07^b^	2.07 ± 0.00^b^	2.02 ± 0.06^b^	1.94 ± 0.16^b^
TAA	36.82 ± 0.90^b^	37.55 ± 0.07^ab^	38.56 ± 0.33^a^	37.57 ± 0.05^ab^	38.15 ± 0.16^ab^	38.31 ± 0.46^a^	38.53 ± 0.94^a^
NEAA	25.94 ± 0.62^a^	24.33 ± 0.05^b^	25.28 ± 0.23^ab^	24.06 ± 0.47^b^	24.93 ± 0.23^ab^	24.88 ± 0.64^ab^	24.60 ± 1.00^ab^
EAA	10.88 ± 0.27^c^	13.23 ± 0.12^b^	13.28 ± 0.10^b^	13.50 ± 0.42^ab^	13.22 ± 0.07^b^	13.43 ± 0.18^ab^	13.92 ± 0.06^a^
EAA/NEAA	0.42 ± 0.00^b^	0.54 ± 0.01^a^	0.53 ± 0.00^a^	0.56 ± 0.03^a^	0.53 ± 0.01^a^	0.54 ± 0.02^a^	0.57 ± 0.03^a^
EAA/TAA	0.30 ± 0.00^b^	0.35 ± 0.00^a^	0.34 ± 0.00^a^	0.36 ± 0.01^a^	0.35 ± 0.00^a^	0.35 ± 0.01^a^	0.36 ± 0.01^a^

^&^*EAA: Essential amino acid; NEAA: Non essential amino acid; TAA: Total amino acid. Differences were analyzed by the Duncan test. Groups without the same superscript letters were statistically different (P <0.05)*.

Isoflavones are a large and very distinctive subgroup of flavonoids that are commonly found in soybeans (40), and they have a significant correlation with the germination of soybeans, which has not been thoroughly studied yet. Vermont *et al*. observed an increase of 16.2% in soybean isoflavones following 18 h of germination (16). Genistin accounted for the largest proportion (40-60%) of soybean isoflavones during 0–36 h, followed by genistein and daidzein (Figure 3C). During soybean germination, the contents of genistein and daidzein were significantly increased. Sharma (41) reported an increasing trend of isoflavones in chickpea (*Cicer arietinum*) during germination, which is consistent with our findings. The observed decrease in the content of isoflavones could be due to the conversion of one type of isoflavones into other types of isoflavones. Terrence (42) reported that soybean primary leaf tissues underwent a programmed shift from isoflavonoid to flavonoid metabolism 3 days after germination. After 36 h, genistein became the highest soybean isoflavone component. An increase in total isoflavones in small-seeded soybeans after 7 days of germination under light conditions has been reported (19). Lee et al. (43) found the contents of phenols and flavonoids dramatically increase in black soybeans germinated for 5 days and may contribute to enhancing antioxidant activity. For the elimination of protease inhibitors, 18 h of germination is recommended (16).

In conclusion, germinated beans are better materials to prepare health products than non-germinated beans. In addition, the improvement of bioactive compounds of soybeans depends on the timing of germination. Soybeans were germinated for 24 h, the bud length was about 0.5–1 cm, and the seeds' protein and isoflavone contents were at their peak. At this stage, germinated soybeans are suitable for the hydrolysis of protein.

### The Results of RSM

**Figure 4:**
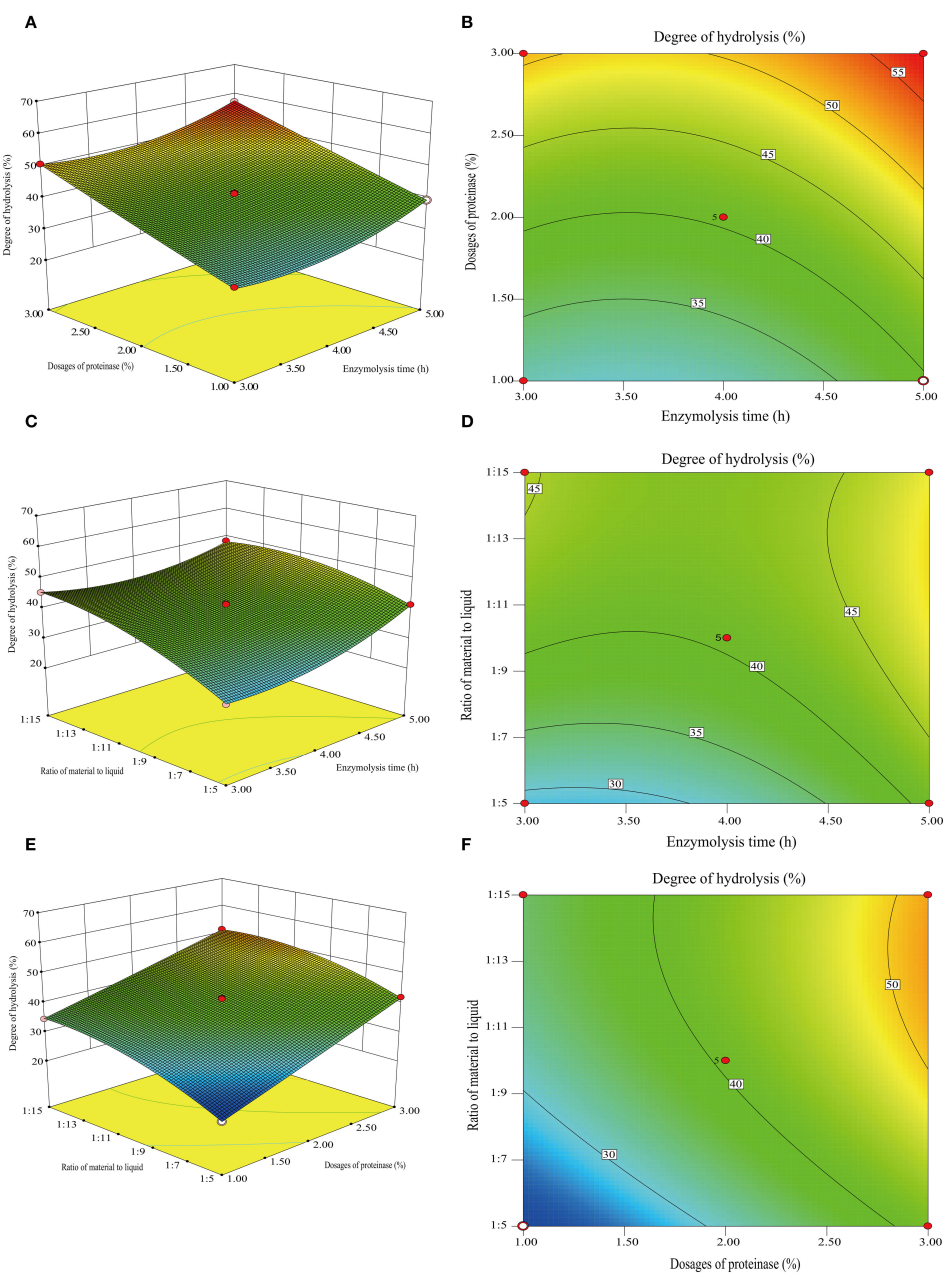
3D response surface plots and 2D contour of the interactive effects on the degree of hydrolysis. Dosages of proteinase and enzymolysis time **(A,B)**, the ratio of material to liquid and enzymolysis time **(C,D)**, and the ratio for material and liquid **(E,F)**.

The effects of three variables, namely, the enzymolysis time (X_1_), dosages of proteinase (X_2_), and the ratio of material to liquid (X_3_), were studied to optimize the hydrolysis conditions using Box-Behnken design and DH as an indicator. The results of the analysis for hydrolysis are presented in Table 1. By comparing the F-value of each factor, it can be concluded that the dosage of proteinase is the most influential factor for DH, followed by the material-liquid ratio and enzymolysis time. A three-dimensional response surface diagram and a contour plot (Figure 4) were plotted based on changes in any two independent variables, which shows the influence of two-factor interaction on the DH of GSPH. As shown in Figure 4, the DH of GSPH gradually increased with an increase in the quantity of proteinase. Figures 4A,E show that the slope of the response surface along the axis of dosages of proteinase was steep, indicating its more influence on the DH of GSPH compared to enzymolysis time and the material-liquid ratio. Figure 4C showed that the slope of the response surface along the axis of the material-liquid ratio was steep, indicating its significant influence on the DH of GSPH.

Based on data analysis using design-export 8.0.6 software, the maximum predictive value of the DH of GSPH was 57.46% when the enzymolysis time was 4.98 h, the dosage of proteinase was 2.95%, and the ratio of material to liquid was 1:11.87. The optimal values of the selected parameters were further subjected to a verification test. The DH of GSPH in this study was (56.49 ± 0.25)%, which was not significantly different from the value of DH 57.46% predicted by the model. There was an increase in the DH following optimization, and the value reached 56.49%. The model established in this study could accurately and reliably predict the DH of GSPH.

### Analysis of Separation Components by Ultrafiltration

#### Molecular Weight Distribution

The results showed that the molecular weight of 97.73% fractions of GSPH was below 3 kDa (Table 3). The GSPH fractions that passed through the 3 kDa ultrafiltration membrane were collected. After ultrafiltration, the GSPH fractions were freeze-dried and collected for further separation. The molecular weights of NSPH (derived from soybean meal protein after oil extraction) and UGSPH are shown in Table 3. It was found that the range of molecular weight of NSPH was broad (82.10–14,860.62 Da) while the molecular weight range of GSPH prepared by the above process was relatively narrow (32.76–8,766.13 Da). By measuring the molecular weight, we found that the molecular weight of GSPH was lower than NSPH, with most of them weighing <3 kDa (97.73%). Furthermore, the proportion of GSPH with a molecular weight ranging from 120 to 400 Da was about 60%, which was higher than that of NSPH (about 45%), which indicates that enzymatic hydrolysis as a part of the above-mentioned protocol was better than that of the protocol used to prepare NSPH, and GSPH generated after enzymatic hydrolysis had a more narrow molecular weight distribution.

**Table 3:** Molecular weight of several soybean protein hydrolysates.

**Samples**	**Peak**	**Retention time (min)**	**Range of molecular weight (Da)**	**Content (%)**
NSPH	1	14.713	3239.34–14860.62	5.12
	2	17.115	943.67–3239.34	15.47
	3	18.063	425.41–943.68	30.29
	4	19.548	134.27–425.41	45.15
	5	21.47	82.10–134.27	3.98
GSPH	1	13.94	3664.73–8766.13	2.28
	2	18.45	411.94–3664.73	31.18
	3	20.09	129.74–411.94	59.55
	4	21.54	81.71–129.74	5.56
	5	22.77	32.76–59.61	1.44
UGSPH	1	18.556	412.38–2803.56	20.77
	2	20.109	130.16–412.38	66.84
	3	21.553	76.74–130.16	9.46
	4	22.665	29.02–63.20	2.93

#### Comparative Analysis of the Antioxidant Activity *in vitro*

*In vitro* antioxidant activities of NSPH, GSPH, and UGSPH were determined using the ferric ion reduction method (FRAP), ABTS^•+^ cation scavenging assay, and DPPH free radical scavenging assay. GSH was used as a positive control. As shown in Figure 2, the antioxidant capacity of GSPH and GSH increased with an increase in concentration. At the same concentration of samples, the total antioxidant capacity of NSPH was significantly lower than the total antioxidant capacity of GSPH, which only increased slightly after ultrafiltration. Carciochi et al. also noticed (44) that the germination process significantly increased the antioxidant activity of quinoa seeds, reaching a greater than the 2-fold increase at 72 h of process in comparison to raw quinoa for the three tested methods. Vernaza et al. (35) observed the highest antioxidant activity in a combination of 72 h of germination and 1 h of Alcalase when compared to the ungerminated and non-hydrolyzed Brazilian soybean flours. Germination can lead to the production of bioactive compounds with potent antioxidant properties. It can modify the concentrations of bioactive compounds of the Brazilian soybean cultivar BRS 133 by increasing lunasin, isoflavone aglycones, and soy saponins (45). However, Shohag et al. (46) reported that hydrophilic antioxidant capacity in soybean and mung bean seeds decreased with germination time (2–10 days). The result may be due to the decrease in phenolic substance content due to the activation of hydrolase and polyphenol oxidase activities during germination (47). The difference may be related to the time of germination. Concentrations with scavenging rates above 70% were selected for discussion. In the ABTS^•+^ free radical scavenging experiment (an electron transfer-based assay), the scavenging rate only showed a slight change in GSPH before and after ultrafiltration. When the concentration was 20.00 mg/ml, the scavenging rate of GSPH reached 89.76%, the same as that of GSH, while the scavenging rate of NSPH was almost zero at the same concentration. As shown in Figure 2C, among the three soybean protein hydrolysates (GSPH, NSPH, and UGSPH), UGSPH had the strongest DPPH radical scavenging ability, and its scavenging rate increased gradually with an increase in mass concentration. When the concentration of NSPH was 20.00 mg/ml, its scavenging rate was 45.28%. While the scavenging rate of GSPH was 77.67% at 20.00 mg/ml, the scavenging rate of UGSPH was 83.02% at 10 mg/ml. However, the concentration is much higher than what is used in many studies. Different materials and methods of hydrolysis may cause this result. In conclusion, the antioxidant activity of UGSPH was much higher than that of NSPH and GSPH as evidenced from these *in vitro* trials. Therefore, we selected UGSPH for further investigation.

### Analysis of Components Separated by Gel Filtration Chromatography

#### Molecular Weight Distribution

**Figure 5:**
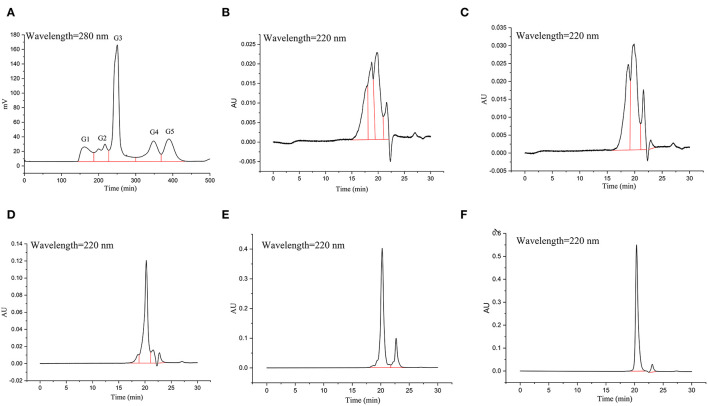
Elution profile and molecular weight of the fraction isolated from UGSPH. **(A)** The germinated soybean peptide was isolated using gel filtration chromatography on Sephadex G-15. **(B)** G1, fraction 1; **(C)** G2, fraction 2; **(D)** G3, fraction 3; **(E)** G4, fraction 4; and **(F)** G5, fraction 5.

The GSPH fraction, which showed the strongest antioxidant activity after ultrafiltration (i.e., UGSPH), was separated by GFC. As shown in Figure 5A, five components, named G1–G5 according to the elution order, were separated. The components were collected and freeze-dried for *in vitro* molecular weight determination and antioxidant activity analysis. The molecular weight distribution of the five components obtained by GFC was determined (Figures 5B–F). Previous studies have shown that antioxidant peptides generally have 2–9 amino acids and have a molecular weight mainly in the range of 400–1,000 Da (48). As shown in Table 4, the molecular weights of both G3 and G4 were <1 kDa and the molecular weight distribution was relatively narrow (about 85.00% of peptides were <1 kDa), while the molecular weight of G5 was relatively small (94.82% of peptides were in 87.37–361.07 Da).

**Table 4:** Molecular weight of fractions of ultrafiltration germinated soybean protein hydrolysates.

**Fractions**	**Peak**	**Retention time (min)**	**Range of molecular weight (Da)**	**Content (%)**
G1	1	18.00	638.46–2734.94	22.93
	2	18.76	365.28–638.46	26.43
	3	19.78	135.12–365.28	41.05
	4	21.61	73.76–135.12	9.60
G2	1	18.82	336.79–1659.03	36.13
	2	19.90	129.82–336.79	48.13
	3	21.60	70.38–129.82	13.78
	4	22.93	31.86–59.93	1.96
G3	1	18.65	394.51–890.97	5.84
	2	20.25	123.93–394.51	80.49
	3	21.57	74.31–123.93	8.95
	4	22.73	33.38–62.31	4.72
G4	1	19.53	285.85–642.86	3.54
	2	20.25	113.12–285.85	80.30
	3	22.70	29.72–89.80	16.16
G5	1	20.35	87.37–361.07	94.82
	2	23.10	43.83–60.59	5.18

#### Comparative Analysis of the Antioxidant Activity *in vitro*

**Figure 6:**
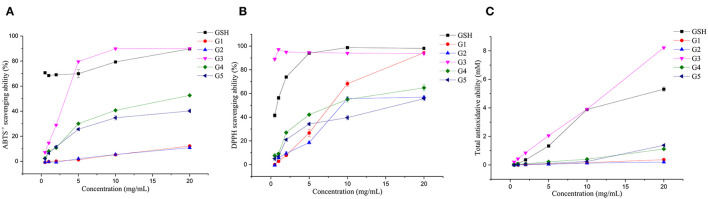
Antioxidative capacities of the fractions isolated from UGSPH. ABTS^•+^ scavenging ability **(A)**, DPPH scavenging ability **(B)**, and total antioxidant ability **(C)**.

The antioxidant activities of the five isolated components were analyzed and compared *in vitro* (Figure 6). G3 had the strongest total antioxidant capacity, which reached 8.22 mM when the concentration was 20.00 mg/ml, which was higher than the total antioxidant capacity of GSH. In addition, the total antioxidant capacity values of G4 and G5 (1.12 and 1.38 mM) were higher than that of G1 and G2 (0.37 and 0.20 mM). This result indicated that the low molecular weight peptides possessed a better radical scavenging ability than the high molecular weight peptides, which corroborated the findings obtained in the germinated foxtail millet (*Setaria italica*) proteins (49). The total antioxidant activity of white quinoa and black quinoa germinated for 24 h was 8.49 and 10.82 mM, respectively (50). Concentrations with scavenging rates above 70% were selected for discussion. In the range of 5.00–20.00 mg/ml of G1–G5, the ABTS^•+^ free radical scavenging rate of G3 was the highest, falling in the range of 79.56 (5.00 mg/ml) to 90.03% (20.00 mg/ml), reaching the level of the scavenging rate of GSH. In the DPPH free radical scavenging experiment, the scavenging ability of G3 proved to be the highest compared to all components. When the mass concentration values were 0.50 and 1.00 mg/ml, the scavenging rate reached 88.99 and 97.16%, respectively, higher than that of the positive control group. It was also higher than the reported DPPH free radical scavenging rate (35.83%) of the hydrolysate obtained from pepsin-hydrolyzed tuna skeleton (the pepsin concentration was 1.30 mg/ml) (51). Notably, buckwheat germinated for 72 h possesses the highest free radical scavenging activity against DPPH (24.50 mM) and ABTS^•+^ (46.01 mM). In contrast, the buckwheat germinated for only 24 h had free radical scavenging capacities of 6.70 and 19.88 mM against DPPH and ABTS^•+^, respectively (52).

## Conclusion and Prospects

Germinated soybeans were used as a raw material for the generation of antioxidative hydrolysates, whereas we determined the optimal state of soybean germination based on several criteria, including the content of moisture, proteins, amino acids, isoflavones, and antioxidant activity. The results showed that protein and isoflavone contents were high in soybean at 24 h following germination (the bud was about 0.5–1 cm). This suggests that germination for a short time can effectively improve the protein and isoflavones of soybean. Five components of UGSPH were separated using GFC. We concluded that GSPH possessed higher antioxidant activity and more concentrated molecular weight distribution, compared to the protein hydrolysates derived from ungerminated soybeans. The molecular weights of G3, G4, and G5 were <1 kDa, whereas G3 showed the strongest total antioxidant capacity and effectively scavenged ABTS^•+^ free radicals and DPPH-free radicals, showing good antioxidant activity. Hence, we conclude that germination is a great process for hydrolyzing soybean proteins and generating GSPH with high antioxidative activities, probably owing to their low molecular weights compared to the hydrolysates generated from ungerminated soybeans.

The high antioxidative activity of G3 that was generated after a short period of germination makes it a potential antioxidative hydrolysate for functional foods. This study lays a foundation for further research on GSPH as potential ingredients for the development of functional foods. The findings give the idea of exploring and utilizing plant seeds as potential resources of biologically active peptides; however, attention should be given to several criteria involved in generating desirable peptides as included and discussed in our trial.

## Data Availability Statement

The original contributions presented in the study are included in the article/supplementary material, further inquiries can be directed to the corresponding author/s.

## Author Contributions

QQ and WW conceived and advised on all aspects of the study. QQ, YZ, and XL performed experiments, analyzed data, and wrote the manuscript. GZ and WW supervised all aspects of the study. QC and FS completed language changes. QX, XW, and YL put forward the advice. All authors discussed and commented on the manuscript.

## Funding

This study was financially supported by the Key R & D project of Zhejiang Province (No. 2018C02048), Zhejiang Basic Public Welfare Research Project (LGN21C200006), Zhejiang Provincial Natural Sciences Foundation of China (under Grant No. LZ22C200006), and Top young talents of the ten thousand talents program of Zhejiang Province (ZJWR0308016).

## Conflict of Interest

The authors declare that the research was conducted in the absence of any commercial or financial relationships that could be construed as a potential conflict of interest.

## Publisher's Note

All claims expressed in this article are solely those of the authors and do not necessarily represent those of their affiliated organizations, or those of the publisher, the editors and the reviewers. Any product that may be evaluated in this article, or claim that may be made by its manufacturer, is not guaranteed or endorsed by the publisher.
